# Empowering Students with Learning Disabilities: Examining Serious Digital Games’ Potential for Performance and Motivation in Math Education

**DOI:** 10.3390/bs15030282

**Published:** 2025-02-27

**Authors:** Georgios Polydoros, Alexandros-Stamatios Antoniou

**Affiliations:** 1 Department of Mathematics & Applied Mathematics, University of Crete, Voutes Campus, 70013 Heraklion, Greece; 2 Department of Pedagogy and Primary Education, National and Kapodistrian University of Athens, 10680 Athens, Greece; as_antoniou@primedu.uoa.gr

**Keywords:** digital serious educational games (SEGs), mathematics performance, learning difficulties, student motivation, COVID-19 impact

## Abstract

This research investigates the impact of digital math serious educational games (SEGs) on enhancing math skills and motivation, specifically focusing on first-degree equations for students with learning difficulties. A comparative study was conducted among two groups of students with learning disabilities. One group engaged with the digital math serious educational game “Battleship”, while the other received traditional curriculum-based instruction. The study’s dual objectives were to assess the effectiveness of digital math SEGs in improving mathematical performance and to evaluate motivation levels. Additionally, gender differences in performance and motivation were examined to understand how SEGs impact boys and girls differently. Employing an empirical approach, a test comprising standard exercises on first-degree equations, typically encountered in seventh grade, was administered. The Motivated Strategies for Learning Questionnaire (MSLQ) was administered to measure motivation. The research sample consisted of 104 seventh-grade students with learning disabilities, aged 12–13 years, from four public schools in the northern sector of Attica, Greece. The sample was evenly divided into two groups of 52 students each. Data were analyzed using SPSS and Excel. Results indicated that students who engaged with the digital math SEG demonstrated significantly improved math performance compared to their peers who used the standard curriculum book. Interestingly, the control group, which used the standard curriculum book, reported higher levels of motivation, underscoring the complex interplay of motivational factors among students with learning disabilities. Furthermore, the analysis by gender revealed that both boys and girls benefited from SEGs in terms of performance. However, motivation levels were only slightly affected by gender, highlighting the potential of SEGs to support diverse learners.

## 1. Introduction

The effectiveness of digital serious educational games (SEGs) in elementary-level mathematics has garnered considerable attention in recent years as educators explore innovative ways to engage students and improve learning outcomes. Research suggests that SEGs not only enhance the enjoyment of learning but also foster critical thinking and problem-solving skills, thereby deepening students’ understanding of mathematical concepts (Lin & Cheng, 2022). Consequently, many schools are increasingly incorporating SEGs into their curricula, offering interactive experiences that cater to diverse learning styles and promote collaboration among peers (Tepho & Srisawasdi, 2023). These platforms often employ gamification strategies that motivate students to persist through challenging tasks and encourage a growth mindset in mathematics. This trend reflects a broader shift in education, where technology and creativity converge to create dynamic learning environments that prepare students for the complexities of the modern world.

This study focuses on investigating the impact of the COVID-19 pandemic on the motivation and performance of students with learning difficulties in mathematics, specifically through the use of a digital SEG with seventh-grade students. The pandemic has significantly affected student engagement, particularly for students with learning disabilities who faced challenges in maintaining motivation and academic performance during the shift to online learning (Alqahtani & Kamhi, 2024; Syakur et al., 2023). While digital tools like SEGs offer interactive and engaging learning experiences, their specific effectiveness in boosting both motivation and performance for students with learning disabilities remains an area that warrants further investigation.

The necessity of this study lies in addressing the generalizability issues associated with the use of game-based learning (GBL) in math education. As prior research has highlighted, the effects of GBL on learning outcomes and motivation are not universally consistent across different settings and populations. This inconsistency may arise from various factors influencing the effectiveness of educational games, such as individual student characteristics, learning environment variations, and content differences (Pan et al., 2022; Schrader, 2022).

This research seeks to bridge this gap by examining how a digital SEG can mitigate the negative effects of the pandemic on academic outcomes and motivation among students with learning difficulties, thereby contributing to the expanding body of knowledge on gamification and digital tools in special education contexts (Tepho & Srisawasdi, 2023). Findings from a systematic review by Dan et al. (2024) also underscore the gaps in the existing literature on primary school-level interventions, further supporting the need for this study.

This research examines whether digital math SEGs contribute to students’ acquisition of knowledge, skills, and motivation in mathematics, particularly in first-degree equations. This will be tested by comparing a group of students who practiced with the digital math SEG “Battleship” against a group of students who studied the standard curriculum book. Thus, the general objective of the research is to determine the effect of digital math SEGs on the math performance and motivation of seventh-grade students (12–13 years old) after the COVID-19 pandemic.

The two specific objectives of the research are the following:

1. To determine whether digital math SEGs improve math performance.
2. To examine students’ motivation after using digital math SEGs.

The three research questions for this study are as follows:

1. Does the use of digital math serious educational games (SEGs) improve the mathematics performance of seventh-grade students with learning difficulties, particularly in first-degree equations?
2. How does the use of digital math SEGs impact the motivation of seventh-grade students with learning difficulties in mathematics after the COVID-19 pandemic?
3. Does gender moderate the relationship between motivation and math performance in students using digital serious educational games (SEGs) compared to those receiving traditional curriculum-based instruction?

These questions aim to investigate both the academic and motivational effects of using SEGs to support students with learning difficulties in the context of mathematics, following the disruptions caused by the COVID-19 pandemic. In addition to its theoretical relevance, this study has practical implications for educational settings by offering an innovative approach to enhance math skills among students with learning disabilities, potentially informing future curriculum designs and intervention programs.

## 2. Literature Review

### 2.1. Digital SEGs on Mathematical Understanding and Student Motivation

Digital SEGs have emerged as a highly effective educational tool, especially in comprehending complex subjects such as mathematics. These interactive games engage students in an immersive learning experience, fostering a deeper understanding of mathematical concepts (Gee, 2003). By allowing active participation and customization to individual learning paces, SEGs provide a personalized learning environment (Anderson & Dill, 2000). This personalization is particularly crucial in catering to diverse learning needs and enhancing overall comprehension. Studies indicate that students using digital educational games show a significant improvement in their mathematical skills, as evidenced by post-test results (Delgado & Mendes, 2023). The interactive nature of these games provides immediate feedback, which is crucial for learning complex mathematical concepts. Gamification elements in SEGs have been shown to stimulate student engagement and motivation, leading to improved performance (Lampropoulos & Sidiropoulos, 2024).

Moreover, SEGs promote experiential learning by encouraging students to experiment with various problem-solving approaches in a risk-free environment (Steinkuehler & Duncan, 2008). This approach can significantly contribute to a comprehensive understanding of mathematical principles. Additionally, research suggests that digital SEGs can enhance memory retention, understanding, and logical reasoning, all of which are essential components of mastering mathematics (Subrahmanyam & Greenfield, 1994).

Recent studies further support these findings. Zainuddin et al. (2020) highlighted that digital SEGs are particularly effective in enhancing mathematical comprehension by providing instant feedback and encouraging active participation. Naul and Liu (2020) demonstrated that incorporating storytelling and narrative elements into SEGs can enhance mathematical understanding by contextualizing abstract concepts. Moreover, Hussein et al. (2022) illustrated how digital SEGs significantly improved students’ problem-solving skills in mathematics. Zhao et al. (2021) showed the positive impact of SEGs on students’ attitudes and motivation towards learning mathematics. Research identifies several reasons why digital SEGs are beneficial for comprehending mathematics, such as the following:

1. Creative learning: SEGs stimulate creative thinking and imagination, enabling players to explore multiple solutions to mathematical problems, thus improving their skills (Anderson & Dill, 2000).
2. Fun learning: the enjoyable nature of SEGs makes learning mathematics a pleasant experience, motivating players to persistently attempt challenges and compete with themselves (Gee, 2003).
3. Experiential learning: SEGs offer an experiential platform where players can immerse themselves in mathematical concepts and apply their knowledge in real-life scenarios, facilitating a deeper level of understanding (Clark et al., 2016).
4. Interactive learning: the interactivity inherent in SEGs engages players actively in the learning process, aiding in the assimilation and comprehension of mathematical principles (Hamari et al., 2014).
5. Social learning: SEGs, whether played individually or in groups, encourage social interaction and collaborative learning, enriching the educational experience (Zheng et al., 2021).
6. Increased motivation: SEGs are designed to offer rewards, incentives, and a sense of progression, effectively enhancing motivation and engagement among learners (Arosquipa Lopez et al., 2023).

### 2.2. Gender Differences

Digital games have emerged as a significant tool in math learning, revealing notable gender differences in engagement and outcomes. Research indicates that, while boys and girls generally perform similarly in digital game-based learning (DGBL), distinct patterns emerge across different grade levels and contexts. Boys typically engage more frequently with video games, which can enhance their digital skills and self-efficacy compared to girls (Scholes et al., 2022). For example, in a study involving primary school learners, girls showed lower self-efficacy in third grade but improved significantly post-intervention, suggesting that DGBL may enhance their cognitive engagement more than boys (Zhang et al., 2024).

Female students also consistently outperformed males in learning outcomes when using specific game features, such as prompted self-explanation, indicating a deeper cognitive engagement with the material (McLaren, 2022; Nguyen et al., 2022). Furthermore, girls exhibited higher intrinsic motivation improvements in DGBL settings, particularly in younger grades, while boys did not show significant changes (Zhang et al., 2024). Female students also engaged less in “gaming the system”, a behavior linked to disengagement, which positively correlated with their learning outcomes (Baker et al., 2024).

Emotional expressions during gameplay revealed that girls experienced more frustration, which could impact their learning experience (Zhang et al., 2024). Conversely, while DGBL shows promise in bridging gender gaps, it is essential to recognize that boys may still engage more with digital games overall, potentially influencing their learning experiences differently (Gunawardhana, 2021).

These findings from prior research not only support the integration of digital SEGs in math education but also provide a robust framework for interpreting our study’s results, which demonstrate significant improvements in math performance when using such digital interventions.

## 3. Methodology

The research is empirical with the questionnaire/test as a tool.

### 3.1. Theoretical Framework

The theoretical framework for this study integrates several educational and psychological theories that support the use of digital educational tools, such as the “Battleship” game, to teach mathematical concepts—specifically first-degree equations. This framework combines cognitive learning theories, motivational theories, and technology’s impact on education. These theories are particularly relevant to understanding the role of serious educational games (SEGs) in promoting student engagement, enhancing motivation, and improving learning outcomes in mathematics (Table 1).

1. Constructivist Learning Theory (Piaget, 1972)

Constructivist theories suggest that learners construct knowledge actively through hands-on interaction with their environment. This approach values problem-solving within meaningful contexts, enabling learners to develop a deeper understanding through personal experience.

2. Motivation Strategies for Learning (Pintrich & de Groot, 1990)

This model posits that motivation plays a central role in shaping learning strategies and academic performance. Motivation influences persistence, effort, and engagement in learning activities. To measure these aspects, this study adapts the Motivated Strategies for Learning Questionnaire (MSLQ), focusing on how digital games versus standard curriculum books affect students’ intrinsic and extrinsic motivation. This tool enables an analysis of whether engaging students through SEGs influences their attitudes and performance in mathematics compared to more traditional methods.

3. Game-Based Learning (GBL) (Gee, 2003)

Game-Based Learning theory highlights how interactive games can be effective educational tools by fostering student motivation and engagement. Games, through elements like challenges, rewards, and feedback, can immerse students in a learning experience that supports active participation and motivation.

### 3.2. Brief Description of the Game

“Battleship” is a free online game developed by Quia, USA (https://www.quia.com/ba/24940.html, accessed on 7 April 2023). The game focuses on helping the player develop skills in solving first-degree equations and has three levels of difficulty. The game starts with a short intro showing a design with five ships. Each time the player chooses a square, so does the opponent. In the event that the selected square belongs to a piece of the enemy’s naval forces, then, depending on the level, a window appears with an equation and certain options. If the player’s answer is correct, then an explosion appears on the selected square: a part that belongs to the naval force has been hit. Otherwise, in the case of an incorrect answer, a target is displayed. The process continues until all of the player’s or the system’s naval forces have been annihilated.

### 3.3. Participants

This study involved 104 seventh-grade students, with 52 students in the digital SEG group—25 boys (48%) and 27 girls (52%)—and 52 students in the standard curriculum book group—24 boys (54%) and 28 girls (46%). These students were selected from four different schools in the northern sector of Attica, Greece, following a positive response from a total of seven schools that were approached through the researchers’ application to the Department of Secondary Education. The selection of students was made purposefully, according to the teachers’ recommendations and their performance on the teachers’ attendance list. During the selection of the students, their prior experience with games was not considered.

To confirm that the sample of 104 students provides the required statistical power, we performed an a priori power analysis using G*Power for an independent samples *t*-test. The parameters were set as follows:

Significance level (α): 0.05;

Desired power (1 − β): 0.80;

Anticipated effect size (Cohen’s d): 0.55.

The following formula was used for calculating the required sample size:

$$n=\frac{2\times \left(Z\left(\alpha /2\right)+Z\left(\beta \right)\right)²}{d²}=\frac{2\times 7.84}{0.3025\;}\approx \;51.84\;\approx \;52$$

where

***Z*_(α/2)_** = 1.96 (for a two-tailed test at α = 0.05),

***Z*_(β)_** = 0.84 (for a power of 80%),

***d*** = 0.55.

Rounding up, each group required approximately 52 participants. Since the study involves two groups, the total required sample size was 52 × 2 = 104 students.

### 3.4. Procedure

To test to what extent the “Battleship” game is more suitable than the standard curriculum book for the acquisition of knowledge related to first-degree equations, an experiment was conducted in a computer room at the participants’ elementary schools. The laboratory was dedicated to this experiment, which took place for two sessions per week after the end of the school day, with the teachers also participating. The students were randomly divided into two groups. The digital SEG group worked on the game on the computer and answered questions from a first-degree equation test formulated by the researchers according to the curriculum. The standard curriculum book group studied the corresponding material from a typical curriculum book and answered the same test as the digital SEG group.

The digital SEG intervention was structured over a period of 6 weeks, with sessions conducted twice a week. Each session lasted approximately 45 min. This schedule was chosen to balance the need for consistent practice with the prevention of cognitive overload. The dosage frequency was selected based on prior research indicating that regular, moderate exposure to digital game-based learning is optimal for enhancing learning outcomes.

To ensure fidelity of implementation, standardized procedures were followed during all sessions. Researchers conducted regular observations, and teachers were briefed and trained on the intervention protocol. This monitoring helped to maintain consistency in the delivery of the digital SEG across different sessions and schools.

All assessments were conducted in a computer lab within the students’ school, with the presence of a teacher or an assistant researcher to ensure a standardized testing environment. Each testing session lasted approximately 45 min, ensuring that the students had adequate time to complete the assessments without experiencing fatigue.

The interval between the pre-test and the post-test was set at the end of sixth week, allowing sufficient time for the intervention to exert its effect while minimizing external influences on performance.

### 3.5. Instruments

The questionnaire consisted of the three following parts (Table 2):

In this study, statements from Part A: Motivation of the Motivated Strategies for Learning Questionnaire (MSLQ) (Pintrich & de Groot, 1990) were used to measure students’ motivation, with adaptations made to translate and contextualize them into Greek. Table 3 resents the adapted statements alongside their original MSLQ equivalents from Part A of the MSLQ.

The specific first-degree equations in parts 1 and 2 were chosen to reflect the typical range of equations seventh-grade students encounter.

A correct answer in part (1) received one point and a wrong answer zero points; the points were added up to give each student’s total score. In part (2), if the students found all the right answers (7 (correct) in total), they received 1 point, 0.5 points if they found 4–6 correct answers, 0.25 points if they found 2 or 3 correct answers, and zero points if they found none or one correct answer only.

Prior to the main study, the instrument—which consisted of three parts, solving equations, checking equivalent equations, and motivational beliefs adapted from the MSLQ—was piloted with a similar cohort to assess its psychometric properties. The pilot study revealed a Cronbach’s alpha of 0.85, indicating strong internal consistency. To further establish content validity, 3 subject matter experts carefully reviewed each item to ensure they accurately captured the constructs of mathematical performance and motivation. Additionally, a Kaiser–Meyer–Olkin (KMO) test was conducted to assess the sampling adequacy for factor analysis, yielding a KMO value of 0.78. This value is considered acceptable, supporting the appropriateness of the instrument for measuring the intended constructs in this study.

### 3.6. Data Analysis

Data analysis was performed using SPPS statistical software (v.24) and the numerical calculations in Excel 10.

## 4. Results

**Figure 1:**
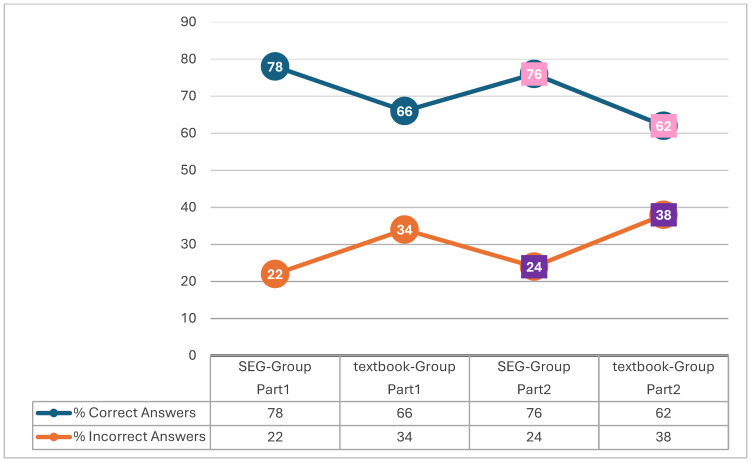
Percentages of correct and incorrect answers of Section 1 and Section 2 for both groups.

Figure 1 shows that the students who studied first-degree equations using the digital SEG solved more exercises (Section 1 and Section 2) correctly than the students who studied first-degree equations from the standard curriculum book. Specifically, in both sections, students who studied first-degree equations using the digital SEG platform exhibited higher performance compared to those who studied through the standard curriculum book. For Section 1, there was a success rate of 78% in the SEG group versus 66% in the standard curriculum book group, and for Section 2, there was a success rate of 76% in the SEG group compared to 62% in the traditional curriculum-based group.

### 4.1. Independent t-Test

According to the results of the independent *t*-test, students in the standard curriculum book group (M = 5.97, SD = 0.11) were more motivated to learn than students in the SEG group (M = 5.18, SD = 0.076), as Table 4 shows. This difference is statistically significant, with a *p* value of 0.00 < 0.05, indicating strong evidence against the null hypothesis. Additionally, Cohen’s d = 2.18 suggests a large effect size, according to Cohen’s (1988) criteria.

### 4.2. Chi-Square

Table 5 shows that for part 1, the *p* value of 0.009 is below the 0.05 threshold, indicating a statistically significant association between the teaching method (SEG vs. standard curriculum book) and the correctness of answers. Similarly, for part 2, the *p* value of 0.023 is also less than 0.05, which further confirms a statistically significant relationship between the teaching method and the correctness of answers in this section as well.

This suggests that the type of teaching method may influence the likelihood of achieving correct answers in both parts.

### 4.3. Regression Model

The regression model presented here aims to understand the influence of gender and motivation on performance. The results highlight that both variables have significant effects, though with differing magnitudes. While gender has a statistically significant but small impact on performance, motivation emerges as a much stronger predictor. The model provides valuable insights into how these factors contribute to explaining performance variance. Below is a detailed breakdown of the regression results (see Table 6).

- Intercept: the baseline performance when both gender and motivation are at zero is 5.10.
- Gender: The coefficient for gender is 0.15, suggesting a small positive effect on performance. However, it remains statistically significant (*p* < 0.05), as indicated by the confidence interval [0.05, 0.25]. This shows that, while small, gender still has a statistically significant influence on performance.
- Motivation: The coefficient for motivation is 0.85, indicating that motivation has a much stronger effect on performance. The confidence interval [0.75, 0.95] is narrow, suggesting high precision in this estimate.
- sr^2^ (squared semi-partial correlation): motivation explains a significant portion of the variance (67%), while gender only explains a small portion (2%).
- r: the correlation for motivation with performance is moderate to strong (r = 0.80), while gender shows a very weak but statistically significant correlation (r = 0.10).
- Fit: the overall model fits well with R^2^ = 0.72, meaning 72% of the variance in performance is explained by both gender and motivation combined.

This model shows that gender has a statistically significant, but minimal, effect on performance. Motivation, however, remains the stronger predictor.

The model is: Performance = 5.10 + 0.15(Gender) + 0.85(Motivation) + ε

**Figure 2:**
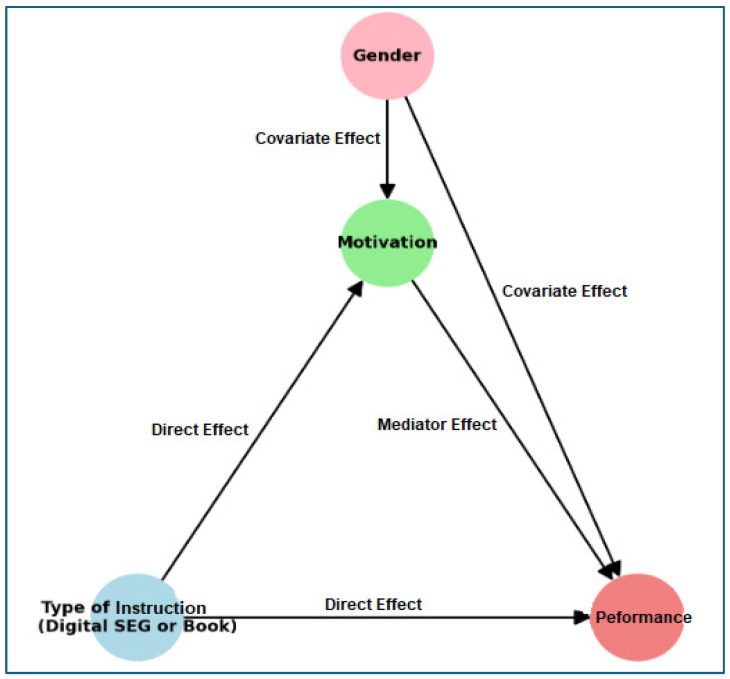
Diagram of motivation, type of instruction, and gender impact on math performance.

The next diagram (Figure 2) reflects the relationships:

- Type of intervention (digital SEG or standard curriculum book) impacts both motivation and performance.
- Motivation is directly linked to performance, indicating that motivation could enhance student outcomes.
- Gender affects both motivation and performance directly.

Figure 2 provides a clearer picture of how instructional type and gender collectively contribute to motivation and math performance, offering a holistic understanding of the influences at play.

## 5. Discussion

### 5.1. Does the Use of Digital Math Serious Educational Games (SEGs) Improve the Mathematics Performance of Seventh-Grade Students with Learning Difficulties, Particularly in First-Degree Equations?

The use of digital SEGs has increasingly been shown to enhance mathematical comprehension by fostering a dynamic, interactive learning environment (Gee, 2003). In this study, students using SEGs to practice first-degree equations outperformed those studying with the traditional standard curriculum book. Specifically, the SEG group achieved a 78% success rate in Section 1 compared to 66% in the standard curriculum book group, and a 76% success rate in Section 2 versus 62% for the standard curriculum book group. These findings are supported by Delgado and Mendes (2023), who reported that digital games significantly improved students’ post-test scores, especially in complex topics like algebra.

The Chi-Square analysis further confirmed a statistically significant association between the teaching method (SEG or the standard curriculum book) and correct answers, underlining SEGs’ efficacy in mathematics. This aligns with Lampropoulos and Sidiropoulos (2024), who found that SEGs provide immediate feedback, something which is critical for grasping challenging concepts, and facilitate personalized learning by adapting to individual students’ needs (Anderson & Dill, 2000). Additionally, Zainuddin et al. (2020) emphasized that SEGs’ narrative elements enhance engagement and aid in the comprehension of abstract concepts by contextualizing them in relatable ways. Steinkuehler and Duncan (2008) also noted that experiential learning through SEGs supports improved problem-solving abilities. Overall, the evidence suggests that digital SEGs significantly impact students’ mathematical performance, particularly for those with learning difficulties.

### 5.2. How Does the Use of Digital Math SEGs Impact the Motivation of Seventh-Grade Students with Learning Difficulties in Mathematics After the COVID-19 Pandemic?

While SEGs are widely recognized for their ability to enhance student engagement through rewards and interactive experiences (Zhao et al., 2021), this study found an interesting contrast: students in the standard curriculum book group reported higher motivation scores (M = 5.97, SD = 0.11) compared to those in the SEG group (M = 5.18, SD = 0.076). This statistically significant difference (*p* < 0.001) with a large effect size (Cohen’s d = 2.18) suggests that the motivational dynamics of traditional learning might differ. Arosquipa Lopez et al. (2023) highlighted that SEGs’ incentive structures can bolster motivation, but these findings raise questions about the contextual factors that might influence motivation, particularly post-pandemic.

Hamari et al. (2014) observed that SEGs facilitate a more interactive, personalized learning experience, which usually translates to higher motivation levels. However, this study suggests that traditional methods might sometimes appeal more strongly to students’ motivational needs. Further, Hussein et al. (2022) found that SEGs boost problem-solving engagement, yet for students recovering from the disruption of COVID-19, familiar standard curriculum book routines may offer comfort and clarity, potentially contributing to higher motivation scores in this context. Thus, while SEGs have clear performance benefits, their impact on motivation may vary based on individual student preferences and external factors, such as the post-pandemic educational landscape.

One possible explanation for the higher motivation scores among curriculum book users is the familiarity and comfort they experience with traditional methods. Given that students have been exposed to conventional teaching throughout their academic careers, the structured and predictable environment of textbook learning may offer a sense of stability—especially in the aftermath of the COVID-19 pandemic.

### 5.3. Does Gender Moderate the Relationship Between Motivation and Math Performance in Students Using Digital SEGs Compared to Traditional Curriculum-Based Learning?

In analyzing the role of gender in the relationship between motivation and performance, the regression results showed that motivation had a more pronounced impact on performance (β = 0.80) than gender (β = 0.10). Motivation explained a significant amount of the variance (sr^2^ = 0.67), while gender’s effect was smaller (sr^2^ = 0.02). These findings align with Scholes et al. (2022), who noted that although boys and girls generally achieve similar performance outcomes in digital learning contexts, engagement patterns and motivational dynamics often differ by gender.

Moreover, Zhang et al. (2024) observed that female students tend to demonstrate greater cognitive engagement in digital learning environments, often benefiting more from game features that encourage self-reflection and problem-solving. McLaren (2022) also highlighted that girls often engage with digital games in ways that improve learning outcomes, such as through prompted self-explanation, which fosters a deeper understanding. Additionally, Nguyen et al. (2022) found that intrinsic motivation levels in digital learning environments tend to be higher among female students, especially in early grade levels. These studies suggest that while gender does play a role in SEG engagement, motivation is the primary driver of performance outcomes, with gender having a more nuanced influence.

Although the analysis indicated that gender had a modest moderating effect on the relationship between motivation and math performance, further investigation is warranted. Future studies could utilize mixed methods approaches—combining quantitative analysis with qualitative methods such as focus groups or in-depth interviews—to explore how gender-specific factors, such as learning preferences or social influences, affect motivation in digital versus traditional learning contexts.

The present findings not only confirm the performance enhancing effects of digital SEGs but also contribute to the theoretical framework underlying game-based learning. By demonstrating a significant improvement in math performance alongside complex motivational dynamics, this study provides empirical support for integrating digital interventions in educational practices. The results offer valuable insights for educators and researchers alike, paving the way for future studies that explore blended learning approaches and long-term retention effects.

Educators may consider integrating digital SEGs as a supplementary tool alongside traditional curriculum-based instruction. For example, a blended approach could involve using digital games to reinforce problem-solving skills and conceptual understanding, followed by classroom discussions or textbook-based exercises that consolidate this knowledge. This approach could cater to different learning preferences and support students with learning disabilities more effectively.

## 6. Limitations and Future Research

This study examined how serious educational games (SEGs) immediately enhance mathematics performance and motivation for students with learning disabilities, yet did not investigate long-term retention or sustained motivation. While SEGs have shown promise in boosting short-term engagement and understanding (Gee, 2003), long-term effects may require more individualized support. Future research should explore whether SEGs provide lasting benefits in both performance and motivation. Additionally, because participation was voluntary, self-selection bias may limit generalizability. Future studies should employ more controlled sampling to better represent the broader student population.

Another important consideration is that potential covariates, such as non-verbal intelligence (IQ) and other intervening variables, were not included in the analysis. This decision was made to maintain a focused examination of the impact of the digital SEG intervention on math performance and motivation. Future research may consider incorporating these variables to further elucidate their moderating effects.

An unexpected finding was that students with learning disabilities using traditional methods reported higher motivation scores than those using SEGs. This raises questions about how recent shifts in learning preferences—possibly influenced by the increase in digital learning during the COVID-19 pandemic—might impact motivation for students with disabilities. Future research could examine whether these observed shifts in motivation reflect a temporary adjustment or a longer-term change in how these students engage with digital versus traditional learning methods.

Although the final sample of 104 students was rigorously selected from an initial pool of 118 eligible participants, it represents only a small fraction of the broader population. This selection was based on strict inclusion criteria, official diagnostic reports from the National Organization, and teachers’ recommendations. While this process ensured a high degree of homogeneity and methodological rigor, future studies should aim to involve a larger and more diverse sample to enhance the generalizability of the findings.

In conclusion, based on the findings, future research should do the following:

- Investigate the long-term effects of digital SEGs on academic performance and motivation through longitudinal studies.
- Include additional covariates, such as non-verbal intelligence (IQ) and socio-economic status, to better understand their moderating effects.
- Explore gender differences in more detail using both quantitative and qualitative methodologies.
- Expand the sample size and diversity to improve the representativeness of the findings.

## 7. Conclusions

This study demonstrated the significant contribution of digital serious educational games in enhancing mathematical performance among students with learning disabilities, particularly in solving first-degree equations. Specifically, students using the digital game “Battleship” achieved higher correct answer rates compared to those who studied using traditional curriculum books.

Despite these performance gains, the curriculum book group reported higher levels of motivation. This discrepancy suggests that while digital games improve performance through interactivity and immediate feedback, traditional learning methods may provide a more familiar and encouraging environment in terms of motivation—especially in a post-COVID-19 context.

Furthermore, the analysis confirmed that motivation is a strong predictor of mathematical performance, whereas gender differences were minimal. This finding supports the idea that digital instructional methods can effectively cater to a diverse range of learners.

Overall, the results advocate for the integration of digital serious educational games into math curricula for students with learning disabilities. However, they also highlight the need for further research to explore the long-term effects of these interventions and to better understand the motivational factors at play. Future studies should investigate blended approaches that combine digital and traditional methods to develop more comprehensive, personalized learning experiences.

## Figures and Tables

**Table 1:** Theoretical framework and application in the Study.

Theory	How It Was Used in the Study
Game-Based Learning (GBL) (Gee, 2003).	The “Battleship” game was used to create an interactive and engaging learning experience where students could practice solving first-degree equations, promoting intrinsic motivation and problem-solving skills.
Constructivist Learning Theory (Piaget, 1972)	The study used the “Battleship” game to provide a hands-on learning experience, where students actively constructed their understanding of first-degree equations through trial, error, and interaction.
Motivation Strategies for Learning (Pintrich & de Groot, 1990)	The adapted MSLQ was used to measure the intrinsic and extrinsic motivation of students in both the digital SEG group and the standard curriculum book group, assessing their engagement and performance in solving equations.

**Table 2:** The questionnaire in the Study.

Part	Tasks	Scoring
(1) Solve the equations	Solve the following first-degree equations: i. 4x = 8 ii. 6 = 2x iii. 5x + 2 + 3x = 12 iv. 14= −7x + 2x − 3 v. 2x: 4 = 3 vi. 3x: 2 − 8: 2 = 0 vii. 9x + 2 − 6 − 5x = 1 viii. 3x + 2 = 4x + 1 ix. −7x +10 = −7x − 10 x. x + 8 = 4x + 4 xi. x/3 = 4/9 xii. x/3 − 10/4 = 1	1 point for each correct answer, 0 for incorrect answers. Total points = sum of correct answers.
(2) Check equivalent equations	Check which of the following equations express the same mathematical relationship as 4x = 8 (do not solve the equations): (1) x = 2 (2) x = 6 − 3 (3) 5x = 10 (4) 7x = 8 (5) x = 12/6 (6) x = 6 + 2 (7) x/3 = 9/2 (8) 12 = 6 + 3x (9) 16 + 7 = 8x + 7 (10) 2x − 10 = 4 − 10 (11) 5x − 7 = 3 (12) 9x − 1 = 18 − 1	Points awarded based on number of correct matches: 1 point for 7 correct answers, 0.5 points for 4–6 correct answers, 0.25 points for 2–3 correct answers, 0 points for 0–1 correct answers.
(3) Motivational beliefs (MSLQ)	Statements from Part A on students’ motivation, adapted from the Motivated Strategies for Learning Questionnaire (MSLQ).	Likert 1–7.

**Table 3:** Statements and their corresponding MSLQ equivalents.

Adapted Statements	MSLQ Equivalent
1. I am motivated to learn mathematics because I believe it is important for my future.	“I believe that I will be able to use what I learn in this course in the future.”
2. I am interested in solving equations, even if they are difficult.	“I find the course material interesting.”
3. I try my best to solve first-degree equations, even if I find them difficult.	“I make an effort to do my best in this course.”
4. I enjoy learning how to solve equations, especially when I understand the concepts behind them.	“I enjoy learning new things in this course.”
5. When I get stuck on an exercise, I try to find a way to solve it instead of giving up.	“When I encounter difficulty in this course, I try to find ways to solve the problem.”
6. I feel confident that I can solve equations correctly.	“I am confident that I will do well in this course.”
7. Solving equations gives me a sense of achievement when I get the right answer.	“I feel a sense of accomplishment when I learn something new in this course.”
8. I try to solve equations by myself without asking for help too quickly.	“I prefer to try and solve problems on my own before seeking help.”
9. I feel that I learn best when I can solve equations in a step-by-step manner.	“I feel that I learn best when I can work through problems systematically.”
10. I believe that learning how to solve equations will help me do well in the future.	“I believe that what I learn in this course will help me in my future career.”

**Table 4:** Independent *t*-test results on students’ motivational beliefs.

Standard Curriculum Book Group		SEG Group		*t*(102)	*p*	Cohen’s d
*M*	*SD*	*M*	*SD*	10.842	0.00 *	2.18
5.97	0.11	5.18	0.076			

Note. * *p* < 0.001.

**Table 5:** Chi-Square analysis.

Variable	*χ* ^2^	*df*	*p*
Part 1—Correct answers	6.78	1	0.009 **
Part 2—Correct answers	5.21	1	0.023 *

Note. * *p* < 0.05, ** *p* < 0.01.

**Table 6:** Regression model.

Predictor	b	b 95% CI [LL, UL]	βeta	βeta 95% CI [LL, UL]	sr^2^	sr^2^ 95% CI [LL, UL]	r	Fit
(Intercept)	5.10 **	[4.00, 6.20]	-	-	-	-	-	-
Gender	0.15 *	[0.05, 0.25]	0.10 *	[0.03, 0.18]	0.02	[0.01, 0.04]	0.10 *	-
Motivation	0.85 **	[0.75, 0.95]	0.80 **	[0.70, 0.90]	0.67	[0.55, 0.75]	0.80 **	R^2^ = 0.72 **

Note. * *p* < 0.05, ** *p* < 0.01. A significant *b*-weight indicates the unstandardized regression coefficients. βeta represents standardized regression weights, sr^2^ indicates the semi-partial correlation squared, and r represents the zero-order correlation. LL and UL represent the lower and upper limits of a 95% confidence interval, respectively.

## Data Availability

The data that support the findings of this study are available upon request from the corresponding author. Due to privacy and ethical restrictions, the data are not publicly available as they contain information that could compromise the privacy of the research participants. As the participants are minors with learning disabilities, access to the data requires obtaining informed consent from all parents before any data can be shared.
